# Comprehensive Investigation of the Differences of the Roots of Wild and Cultivated *Mirabilis himalaica* (Edgew) Heim Based on Macroscopic and Microscopic Identification Using HPLC Fingerprint

**DOI:** 10.1155/2020/8626439

**Published:** 2020-04-10

**Authors:** Yuanyang Shao, Lian Peng, Hui Lin, Jiahui Li, Yuetong Yu, Guangzhao Cao, Huiqin Zou, Yonghong Yan

**Affiliations:** ^1^School of Chinese Materia Medica, Beijing University of Chinese Medicine, Beijing 102488, China; ^2^Department of Pharmacy, The Third Affiliated Hospital, Sun Yat-Sen University, Guang Dong 510630, China; ^3^Department of Pharmacy, Lichuan Ethnic Hospital of Traditional Chinese Medicine, Hubei 445400, China

## Abstract

*Mirabilishimalaica* (Edgew) Heim (*MH*) is an important Tibetan medicine with demonstrated medicinal efficacy and promising developmental value. A previous study of *MH* was limited to vague morphological and microscopic descriptions, restricting its clinical application and further development as a medicine. The goal of this study was to comprehensively characterize wild and cultivated products of *MH* using macroscopic and microscopic identification using HPLC fingerprint. The results revealed that the cultivated and wild *MH* exhibited differences in macroscopic and microscopic characteristics and chemical components. This analysis can facilitate the establishment of a more comprehensive quality evaluation method for *MH*. These results provide the basis for clinical applications and the improvement of quality standards of *MH* as a step towards modernization of Tibetan medicine.

## 1. Introduction

The Qinghai-Tibet Plateau has a unique geographical environment, with high altitude, strong ultraviolet rays, thin air, a large temperature difference between day and night, and many unique species. Many of these unique species have been used as components of Tibetan medicine due to their bioactive effects. However, current Tibetan medicine quality standards have disadvantages, such as backward evaluation technology, a single evaluation component, cumbersome evaluation methods, and incomplete evaluation systems, which seriously restrict development and clinical use. The application of modern scientific tools to investigate these species can provide insight into available medicinal plant germplasm resources and facilitate their eventual development into modern medical products. Tibetan medicine has a long history [1–6]. AshaGanaha is originally from *Mirabilis himalaica* (Edgew) Heim radix (*MH*), and its dried root is popular in Tibetan medicine (TM), a medicine that is used to treat nephritis, edema, gonorrhoea, lumbago, arthralgia, uterine cancer, and bladder stones [7, 8]. The components of *MH* include glycolipids, rotenoids, phenylpropionate derivatives, steroids, glucosides, organic acids, lignin, and anthraquinones [8–11]. Modern pharmacological studies have shown antifertility effects of *MH* seed extract in mice [12]. The *MH* components mirabijalone E, 9-O-methyl-4-hydroxyboeravinone B, boeravinone C, and boeravinone H were isolated from the radix and exhibited toxicity to A549 and HeLa cells [13]. Additionally, (E)-3-(4-hydroxy-2-methoxyphenyl)-propenoic acid 4-hydroxy-3-methoxyphenyl ester was found to have inhibitory activities on tumour growth in vivo using xenografts of HepG2 cells [14]. Due to these important pharmacological effects, wild *MH* resources have been exploited [15]. The plant can be cultivated, but little is known about how the chemical composition and the quality of *MH* cultivars might differ from those of wild plants. Therefore, comprehensive identification methods are urgently needed to allow for the accurate identification of wild and cultivated *MH*. This is an important step required to ensure the safety of clinical medication and will provide scientific support for the future development and utilization of *MH*.

Tibetan medicine is an important part of traditional Chinese medicine (TCM), and the classic medicinal identification methods used for TCM are also applicable to Tibetan medicine. Morphological identification is a classical and frequently applied method of identification of TCM, using “observing,” “touching,” “tasting,” and “smelling” to identify plant materials [16]. Previous studies reported significant differences in morphological characteristics between cultivated and wild products of *Glycyrrhiza uralensis*, *Gentiana macrophylla,* and other medicinal plants, with correlations between their morphological characteristics and their effective components [17–19]. However, there has been little morphological analysis to compare cultivated and wild *MH*.

In addition to direct morphological observation of plants, the internal structures of plants can be analysed using microscopy, which provides more detailed information that can be used to accurately identify medicinal plants from multiple origins or with similar characteristics. For example, microscopy could distinguish differences in the tissue structure and the degree of development of cultivated and wild-grown plant samples of *Salvia miltiorrhiza* Bge and *Fritillaria taipaiensis* P. Y. Li [20–22]. However, the microscopic characteristics of *MH* have not been described.

The authenticity, purity, and quality of plants used for TCM can be evaluated based on physicochemical identification using physical or biochemical analysis methods. High-performance liquid chromatography (HPLC) fingerprint is an effective method that facilitate quality control of traditional medicine materials. HPLC fingerprint studies also revealed significant differences in the characteristics of the effective components of wild and cultivated *Bletilla striata* (Thunb) Reich. F. and *Polygalae Radix* [23,24]. HPLC fingerprint techniques could be developed for quality control in *MH* cultivation and utilization.

In this study, wild and cultivated *MH* were evaluated based on macroscopic and microscopic studies using HPLC fingerprint. Our results provide scientific evidence for the further development and utilization of *MH* for clinical application and offer a reference to guide the modernization of other Tibetan medicines.

## 2. Materials and Methods

### 2.1. Plant Material

Cultivated and wild products of *MH* were collected in Lhasa (91°13′N, 29°65′E, altitude 3658 m), Zada (79°98′N, 31°48′E, altitude 3600 m), Gongga (90°98′N, 29°30′E, altitude 3750 m), Linzhi (94°37′N, 29°68′E, altitude 3000 m), and the Gannan Tibetan Autonomous Prefecture (102°92′N, 34°98′E, altitude 2300 m) (Table 1). The dry roots of *MH* were identified by Prof. Yonghong Yan (Beijing University of Chinese Medicine, Beijing, China).

### 2.2. Morphological Identification

Appearance, cross-sections, odours, and tastes of different batches of *MH* were observed using the traditional morphological identification skills of “observing”, “touching”, “smelling”, and “tasting”.

### 2.3. Microscopic Identification

Paraffin sections and temporary powder sections of cultivated and wild *MH* were prepared, and the characteristics of cultivated and wild products were observed by electron microscopy (Olympus DP71) using traditional methods.

### 2.4. Physicochemical Identification

The optimum conditions for HPLC fingerprint were determined by comparing different parameters, including extraction solvent (methanol, ethanol, and ethyl acetate), column temperature (20°C, 25°C, and 30°C), extraction methods (ultrasound and reflux), injection volume (15 *μ*L, 20 *μ*L, and 25 *μ*L), elution conditions 1: 5%–30% B (0–20 min), 30%–80% B (20–40 min), and 80%–95% B (40–50 min); 2: 5% B (0–5 min), 5%–20% B (5–15 min), and 20%–95% B (15–40 min); 3: 5%–30% B (0–10 min), 30%–80% B (10–40 min), and 80%–95% B (40–42 min); and 4: 5%–20% B (0–10 min), 20%–80% B (10–40 min), and 80%–95% B (40–42 min), and detection wavelength (240 nm, 280 nm, 310 nm, and 360 nm). After optimization, the following optimized conditions for HPLC fingerprint were selected: 30 min of ultrasonic extraction in methanol, an injection volume of 20 *μ*L onto the column (Agilent ZORBAX SB-C18 column with dimensions of 4.6 × 250 mm, 5 *µ*m particle size, Agilent Corp, California, USA), and A (water) and B (acetonitrile) as the mobile phase, with detection at 280 nm and a temperature of 25°C. The gradient elution programme was 5%–20% B (0–10 min), 20%–80% B (10–40 min), and 80%–95% B (40–42 min). The main chemical constituents of wild and cultivated *MH* were investigated, and the fingerprints of the main chemical constituents were established. Multiple analysis methods were applied for multivariate statistical analyses using SIMCA-P version 13.0 software (Umetrics, Umea, Sweden), such as principal component analysis and partial least squares discriminant analysis [25,26].

## 3. Results and Discussion

### 3.1. Morphological Identification

**Figure 1:**
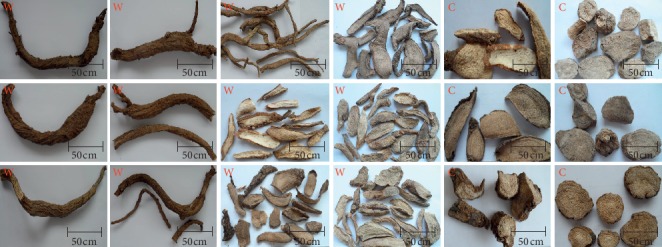
Morphological characteristics of *MH*. W: wild; C: cultivated.

The characteristics of wild and cultivated *MH* were identified by morphological identification as the first step of identification and characterization. The colour of the surface of wild *MH* samples was yellow-brown, and the colour of the cultivated samples was pale yellow to pale brown. The wild *MH* root was yellow-white with clear punctate fringes, and the cultivated root sample was grey-white to grey-brown with indistinct punctate fringes. The root texture of wild *MH* was solid and resistant to breakage, with an intense earthy smell. The root of the cultivated plant was light in texture, easy to break, and had a faint earthy smell. The plant samples shared characteristics of pungency, astringency, and induction of a prickly feeling in the throat after chewing (Figure 1). Overall, there were differences in the characteristics between wild and cultivated species, allowing for the preliminary qualitative identification of wild and cultivated *MH* based on morphological identification.

Due to the serious lack of wild resources, there are a large number of cultivated products of *MH* on the market. But the chemical composition and the quality of *MH* cultivars might differ from those of wild plants. And there were differences in the morphological characteristics between wild and cultivated species, and its incorrect use could result in serious consequences for patients. However, the morphological identification of the wild and cultivated *MH* cannot be achieved according to the current standards [27]. Therefore, the morphological parts of *MH* were supplemented and improved in this section. An accurate morphological identification of *MH* could be achieved based on these characteristics, which could provide a basis for the identification of cultivated and wild *MH* and other TMs in morphological identification [13].

### 3.2. Microscopic Identification

**Figure 2:**
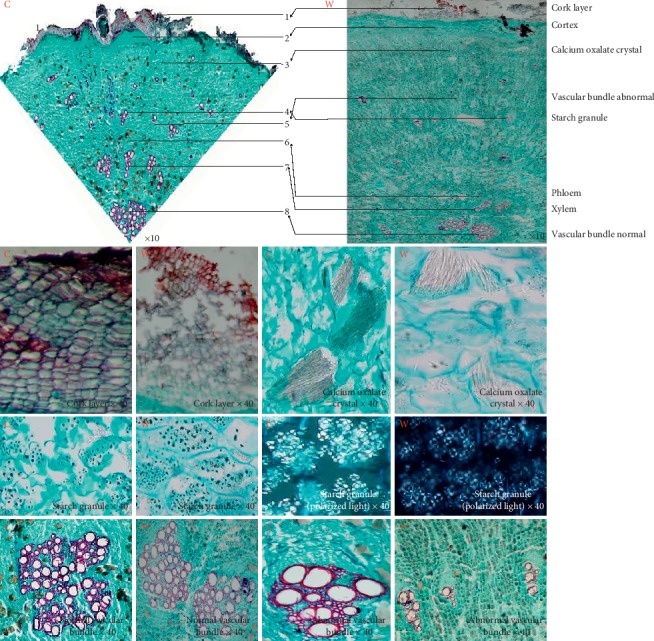
The microscopic characteristics of *MH.* C: cultivated; W: wild.

**Figure 3:**
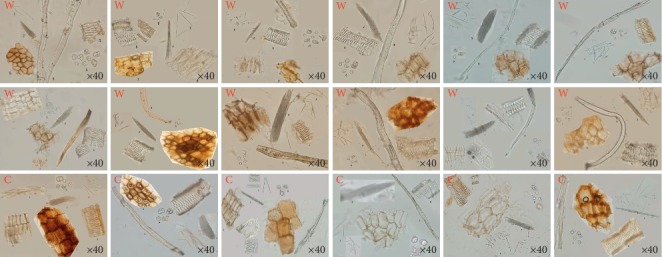
Powder characteristics of *MH.* W: wild; C: cultivated.

Microscopy can be used for more accurate identification of plant medicines. Transverse sections and powder and paraffin sections of cultivated and wild *MH* samples were analysed. The cork layer of the cultivated products appeared flat on the transverse section and that of the wild sample appeared rectangular or square. The cortex of the cultivated species was composed of 12–13 columns with parenchyma cells and that of the wild sample was composed of 8–10 columns and a loose arrangement. The transverse section of the cultivated *MH* contained three to four rounds of abnormal vascular bundles, fewer than the observed four to six rounds in the wild sample. The primary xylem of the normal vascular bundle of the cultivated was tetrarch and that of the wild sample was diarch. Large amounts of raphide and starch grain were observed in the parenchyma cells in both cultivated and wild samples (Figure 2). However, there were no significant differences in the appearance of the powder samples of cultivated and wild products (Figure 3). The cultivated and wild species exhibited differences in the shape of the cork layer, the composition of the cortex, the distribution of abnormal vascular bundles, and the configuration of primary xylem. The cultivated and wild products could be distinguished based on these differences.

There is certain subjectivity in morphological identification. The qualitative identification of *MH*, which relies only on morphological identification without analysis of the internal structure, is not objective, and the microscopic features in the current *MH* quality standards are incomplete. Subsequently, the cultivated and wild *MH* were microscopically studied simultaneously for the first time, and there were differences in the shape of the cork layer, the composition of the cortex, the distribution of abnormal vascular bundles, and the configuration of primary xylem between them. The cork layer of the cultivated and the wild products appeared flat and rectangular or square. The cortex of the cultivated species was closely arranged and that of the wild sample was a loose arrangement. The abnormal vascular bundles of the cultivated *MH* were fewer than those of the wild sample. The primary xylem of the normal vascular bundle of the cultivated and the wild sample was tetrarch and diarch. These differences provided effective data for the supplementation and improvement of microscopic characteristic determination and accurate qualitative identification.

### 3.3. Physicochemical Identification

**Figure 4:**
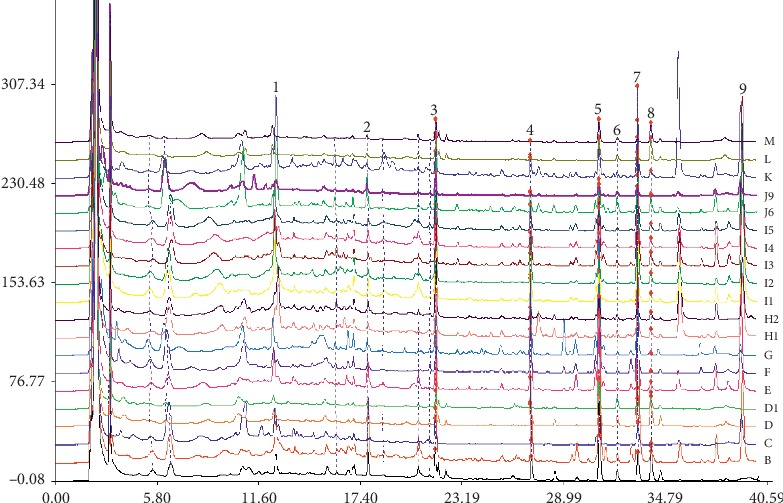
HPLC fingerprint of *MH*. (A, B, C, E, H1, H2, I1, I2, I3, I4, K**)**: wild, **(**D, D1, F, G, J6, J9**)**: cultivated, (4): boeravinone C, (5): boeravinone F, (6): mirabijalone A, (7): mirabijalone I, (8): Abronione, and (9): mirabijalone H.

**Figure 5:**
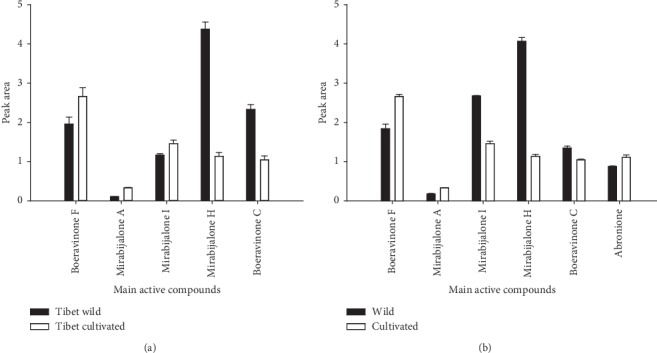
Analysis plot of the peak area for the main active compounds of *MH*. (a) *MH* in Tibet area; (b) *MH* in different producing areas of *MH*.

**Figure 6:**
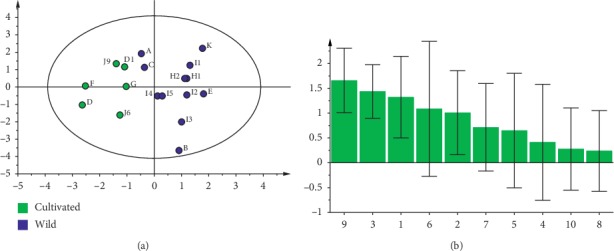
Principal component analysis (R^2^X = 0.548, *Q*^2^ = 0.144) of main chemical components of *MH.*

Among the methods for the quality control of TMs and TCMs, HPLC is one of the most widely used methods. HPLC fingerprint of the active constituents of *MH* was established by optimizing the HPLC detection conditions for the active ingredients of *MH*. The results showed nine peaks in both cultivated and wild products. Peaks 4, 5, 6, 7, 8, and 9 were identified as boeravinone C, boeravinone F, mirabijalone A, mirabijalone I, abronione, and mirabijalone H (Figure 4). The principal component analysis score map suggested that differences in the main active components of *MH* correlate to different growth patterns. The multiple analyses indicated relatively higher amounts of the main chemical components of wild *MH* than those in the cultivated sample. The HPLC fingerprint of *MH* was constructed for the first time, revealing differences in the types and contents of the main components of *MH* with different growth patterns (Figures 5 and 6). The HPLC fingerprint of *MH* established in this study allows for the qualitative and quantitative identification of *MH* with different quality and growth patterns.

It is not enough to rely on qualitative identification to evaluate the pharmacological and medicinal effects of medicinal materials, as the content and identification of active ingredients of medicinal materials are more important. In this study, HPLC fingerprint methods of *MH* analysis were established and optimized for the first time, which provided effective data for the identification and content determination of active ingredients and can be a reference for the study of the content of active ingredients in other Tibetan medicines.

## 4. Conclusions

Tibetan medicines are important traditional medicines with excellent clinical efficacy [28–32]. Among them, *MH* holds an important position. However, the clinical application and comprehensive exploitation of Tibetan medicine are limited due to a lack of systematic research. Moreover, as wild *MH* is going extinct, a quantity of cultivated products has appeared on the market, which may pose a huge threat to human health. For the first time, *MH* was analysed as a representative TM, and relatively comprehensive research on the Tibetan medicine was conducted based on macroscopic characteristics and microscopic characteristics using HPLC fingerprint. These methods can be used to identify this medicinal plant, and this type of comprehensive analysis could be applied to characterize other Tibetan medicines. Our data provide a scientific basis for the evaluation of *MH*, which could facilitate its development as a medicine.

Moreover, to more accurately analyse the macroscopic and microscopic characteristics, determine the identification and content of active ingredients, and evaluate the different accumulated mechanisms of the active ingredients from diverse production areas and different growth patterns of Tibetan medicine, using *MH* as a reference, we will investigate the following: in microscopic identification, the microfeatures and micromorphological characteristics of *MH* will be analysed based on polarizing microscopy, stereo microscopy, and other identification technologies to greatly improve the understanding of the macroscopic and microscopic characteristics of *MH*. For physicochemical identification, ultraperformance liquid chromatography coupled with quadrupole time-of-flight-mass spectrometry, infrared ray-mass spectrometry, high-performance capillary electrophoresis-mass spectrometry, mass spectrometry imaging, and nuclear magnetic resonance will be employed to identify new components and explore the accumulation and structure of active *MH* components. In summary, we will apply the above technologies to perform a more comprehensive study on *MH* and provide better systematic data references for the quality standards and modernization of *MH* and other Tibetan medicines in the future.

## Figures and Tables

**Table 1:** The list of *MH* used in this study.

Sample	Locality	Date of collection	Growth years	Growth patterns
A	Gansu	October 2014	3	Wild
H1	Gansu	October 2014	3	Wild
H2	Gansu	October 2014	3	Wild
B	Lhasa	October 2014	3	Wild
I1	Lhasa	October 2014	3	Wild
I2	Lhasa	October 2014	3	Wild
I3	Lhasa	October 2014	3	Wild
I4	Lhasa	October 2014	3	Wild
I5	Lhasa	October 2014	3	Wild
C	Lhasa	October 2014	3	Wild
K	Tibet	October 2014	3	Wild
E	Tibet	October 2014	3	Wild
D	Tibet	October 2014	3	Cultivated
D1	Tibet	October 2014	3	Cultivated
F	Tibet	October 2014	3	Cultivated
G	Tibet	October 2014	3	Cultivated
J6	Tibet	October 2014	3	Cultivated
J9	Tibet	October 2014	3	Cultivated

## Data Availability

The data used to support the findings of this study are available from the corresponding author upon request.

## Supplementary Materials

Supplementary Materials Primers and reaction system for amplifying the ITS2 sequence of *MH*.
