# Supplementary material for: Comprehensive Investigation of the Differences of the Roots of Wild and Cultivated Mirabilis himalaica (Edgew) Heim Based on Macroscopic and Microscopic Identification Using HPLC Fingerprint
Source: Evid Based Complement Alternat Med. 2020 Apr 10;2020:8626439. doi: 10.1155/2020/8626439 (PMC7171630; doi:10.1155/2020/8626439)
Supplement: Supplementary Materials — Primers and reaction system for amplifying the ITS2 sequence of MH. [file 8626439.f1.pdf]

Supplementary Materials

The primers:

P1:5'-AGAAGTCGTAACAAGTTTCCGTAGG-3',

P4:5'-CCTCCGCTTATTGATATGC-3',

The reaction system:

Prime STAR ® HS Premix 25 μL (purchased from TAKARA Company), P1(1μL) and P4(1μL), DNA(2.5ng), sterilized distilled water(21.5μL);

The amplification procedures: pre-denaturation at 95℃ for 2 minutes, denaturation at 94℃ for 30 s, annealing at 55℃ for 30 s, extension at 72℃ for 80 s, 35 cycles and extension at 72℃ for 10 minutes.
